# Factors influencing the utilisation of Youth Friendly Health Services in Blantyre, Malawi

**DOI:** 10.4102/hsag.v29i0.2411

**Published:** 2024-03-13

**Authors:** Grace C. Sibande, Rakgadi G. Malapela

**Affiliations:** 1 Department of Health Studies, College of Human Sciences, University of South Africa, Pretoria, South Africa

**Keywords:** contraception, factors, influencing, utilisation, youth, Youth Friendly Health Services

## Abstract

**Background:**

Usage of Youth Friendly Health Services (YFHSs) remains unsatisfactory in sub-Saharan Africa despite global agreements on the utilisation of these services among the youths.

**Aim:**

The aim of the study was to identify factors that influence the utilisation of YFHSs in Blantyre, Malawi.

**Setting:**

Four health centres in Blantyre, Malawi.

**Methods:**

A descriptive quantitative research design using multistage sampling was used to randomly sample (*N* = 293) unmarried youths and collect data using a structured questionnaire. Data were analysed using a computerised statistical package for social sciences (SPSS) version 26. Chi-square (χ²) was used to test the significance of the association between variables, and the *p*-value (*p* < 0.05) was considered significant. Regression analysis was used to examine the influence of independent variables on the utilisation of the services.

**Results:**

Less than half of the respondents have ever accessed YFHSs (43%). The Chi-square test showed that the following variables had a significant association with utilisation of the services (*p* < 0.05): gender, age, knowledge, signage, printed health education materials, provider attitudes and being shy or fear of being seen at the services.

**Conclusion:**

Age, knowledge, signpost, printed health education materials, provider attitudes and being shy or fear of being seen at the YFHSs are factors that influenced the utilisation of the services. Working on these factors would help to increase utilisation.

**Contribution:**

The study findings will help to fill the gap in the provision of YFHSs and thus increase utilisation of the services.

## Introduction

Youth Friendly Health Services (YFHSs) were introduced by the World Health Organization (WHO) in 2001 with the purpose of providing acceptable youth services that were nonjudgemental, sensitive, competent and offered with respect and confidentiality (Baroudi et al. 2020). However, utilisation of these services remains low especially in sub-Saharan Africa which includes Malawi (Ninsiima, Chiumia & Ndejjo 2021). According to Ninsiima et al. (2021), youth is a period of optimum health with a series of physiological, psychological and social changes that may expose the youth to unhealthy explorative sexual behaviour. Despite youth being an important age for laying foundations of good health, there exists significant death and illness because of preventable or treatable causes such as early childbearing and unsafe abortions (WHO Regional Office for Africa 2021).

Malawi introduced YFHSs in 2007 to develop interventions that would address the problem of low contraceptive use among teenagers and provide them with a platform where contraceptives could be accessed in a more youth-friendly environment (United States Agency for International Development [USAID] 2014). An evaluation of YFHSs was conducted in 2014 by the USAID in Malawi and the results showed that only 13% of the youths accessed YFHSs (USAID 2014).

Studies conducted in Ethiopia, Ghana and Kenya have also highlighted underutilisation of YFHSs (Amaje et al. 2022; Asare, Aryee & Koto 2020; Embleton et al. 2023; Habtu, Kaba & Mekonnen 2021; Sertsu et al. 2023; Tsegaw, Kassie & Alemnew 2023). Embarrassment in seeking contraceptive services, unfriendliness, judgemental provision of care and lack of confidentiality contributed to young people preferring to utilise providers such as local pharmacies that offer faster and more discreet services than public clinics despite lacking a more comprehensive range of contraceptives (Radovich et al. 2018).

Malawi has a high teenage pregnancy prevalence rate of 29% (Dombola, Manda & Chipeta 2020). This is worrisome because unintended pregnancies can be easily prevented through the youth’s use of contraceptives, which are readily available at YFHSs in Malawi. However, there are possibly many factors that prevent youths from utilising YFHSs in government institutions where contraceptive methods are distributed at no charge. Characteristics of service provision such as acceptance, friendliness, nonjudgemental provision of care and confidentiality are the most important factors in care-seeking behaviours (Radovich et al. 2018). Embarrassment associated with seeking contraceptive services is a common barrier to youths’ utilisation of YFHSs. This results in youths accessing providers like local pharmacies, which offer faster and more discreet services compared to public clinics, with a more comprehensive range of family planning methods. Other characteristics that attract young people to these limited-capacity providers are accessibility, extended opening hours, confidentiality and quick services (Radovich et al. 2018).

A systematic review of factors influencing access to and utilisation of YFHSs in sub-Saharan Africa revealed either structural or individual barriers (Ninsiima et al. 2021). Structural barriers included inconvenient operating hours, health workers’ negative attitudes and unskilled health workers. Some health workers were reported as using abusive language, while others lacked the sympathy expected of service providers. It emerged that some health workers were partially trained to deliver services to young people, while others were entirely untrained. Individual barriers also included a lack of knowledge among youths regarding YFHS issues. Access to reproductive health information was also often hindered because of stigma related to the youths’ age and parental consent (Ninsiima et al. 2021).

According to Kiani, Ghazanfarour and Saeidi (2019), pregnancy and childbirth complications are the leading causes of death among 15–19-year-old girls globally; an additional 3.9 million unsafe abortions occur each year among girls aged 15 years – 19 years, contributing to maternal mortality and complications. This is also the case in Malawi where it is claimed that large numbers of maternal deaths in the country result from illegal abortions (Odland et al. 2018). According to the WHO (2022), prevention of pregnancy and its associated complications including pregnancy-related mortality and morbidity among adolescents are crucial for the attainment of positive health outcomes across the life path as well as being necessary for the achievement of the maternal and newborn health Sustainable Development Goals. While interventions have been introduced to improve Malawi’s situation, evidence that YFHS uptake has improved in the country is inadequate. The purpose of the study was therefore to identify factors that influence the utilisation of YFHSs in Blantyre, Malawi.

This study was therefore deemed necessary as it was envisaged by the researcher that the study findings and recommendations would potentially assist in increasing utilisation of YFHSs. By extension, this should increase the uptake of contraceptives which would in turn reduce the numbers of unintended pregnancies among the youth, unsafe abortions, and the associated complications which can include disability and maternal death (Cameron 2018).

The Health Belief Model was the underlying theoretical basis for this study. Polit and Beck (2021) describe Becker’s 1978 Health Belief Model as one which provides a behavioural explanation of people’s compliance with healthcare usage. This Model is founded on two components: firstly, people will take such actions as they see fit to prevent poor health, and secondly, people will seek healthcare based on the belief that the particular action they have chosen will prevent poor health (Polit & Beck 2017). The model is composed of six constructs to health-seeking behaviour: the perceived benefits, perceived barriers to access, perceived susceptibility, perceived severity, cue to action and self-efficacy. The same model was also successfully applied by Alagrisamy and Arokiasamy (2019) in their study on the uptake of contraceptives by young rural women in Malaysia.

## Aim of the study

The aim of the study was to identify factors that influence the utilisation of YFHSs in Blantyre, Malawi.

### Specific objectives

- Determine accessibility of YFHSs.
- Assess youth knowledge about YFHSs and sexual reproductive health (SRH).
- Determine experiences of the youth at YFHSs.
- Identify barriers to the utilisation of YFHSs.

## Research methods and design

### Study design

A descriptive quantitative research design was chosen for this study using a survey. This methodology was chosen. The descriptive research design was chosen because of its descriptive nature that allowed the researcher to explain the phenomena under study in depth (Wings 2021). In a quantitative research design, different variables are examined while using numbers and statistics to analyse their findings. This method focuses on objectivity and is appropriate when collecting quantifiable measures of variables and inferences from samples of a population.

### Study setting

The study was conducted in four health centres and their surrounding catchment areas. Health centres offer ambulatory and maternity services. They are meant to serve an average population of 10 000 people; however, some urban facilities serve up to 237 000 people and are staffed by mid-level practitioners including nurses, medical assistants or clinical officers. The clinician and nursing roles at the health centres are largely curative, with minimal health promotion and preventive responsibilities (Makwero 2018). The selected health centres included two from Blantyre urban, namely Ndirande and Chilomoni, and two were from Blantyre rural, namely Mdeka and Madziabango. These health centres were identified through a multistage sampling method which was used to include youths from a wider area of Blantyre and from both rural and urban areas.

### Study population and sampling

The study population included young people aged 10 years – 24 years. The WHO defines adolescents as individuals aged 10 years – 19 years; youth as individuals aged 15 years – 24 years and young people as individuals aged 10 years – 24 years (WHO 2023). The study used the age group of 10 years – 24 years because YFHSs are patronised by both adolescents and youth according to the WHO definitions. In this study, young people were used interchangeably as youths. The inclusion criteria for this study were boys and girls aged 10 years – 24 years, who were not married, who were willing to participate in the study, who provided consent or assent to participate in the study and who were able to speak English or the national local language. The exclusion criteria were boys or girls who were not residents of the selected study sites.

A multistage sampling technique was used to generate the sample. Consequently, the Blantyre disctrict was first divided into two clusters, Blantyre urban and Blantyre rural. Blantyre urban has six public health centres from which two were selected using simple random sampling. Blantyre rural, on the other hand, has eight traditional authorities (TAs), which were grouped into two clusters, North and South. Two TAs, one from the Northern cluster and the other from the Southern cluster, were then selected using simple random sampling (Sibande & Malapela 2023). Continuing with simple random sampling, one health centre was selected from the Northern cluster’s three health centres and one from the two centres in the Southern cluster. In total, therefore, four health centres were selected: two from Blantyre urban and two from Blantyre rural (Sibande & Malapela 2023).

The respondents were recruited from the health centres among youths who came to the health centres for YFHSs and other services and from the households in the surrounding catchment areas. With the assistance of the YFHSs, focal persons’ information was given to the youth about the data collection prior to the data collection process. On data collection day, the aim of the study was introduced to the respondents individually to give them an overview of the study and build rapport prior to actual data collection, and consent was sought. Informed consent forms were signed by all participants, parents of youth below 18 years and assent forms for youths below 18 years to prove that no one had been coerced into participating. Data were collected in 10 days.

The required sample size was determined by using the Leslie Kish sampling formula. This formula was preferred as it factors in the level of precision, the 95% level of confidence or risk and the degree of variability in the attributes being measured. The formula is shown as follows:

$$n=\frac{z{\left(\alpha /2\right)}^{2}p(1-p)}{d^{2}}$$ [Eqn 1]

Where *n* = sample size,

*z* (α/2)^2^ = confidence interval,

*p* = proportion of utilisation of Youth Friendly Health Services in Malawi (USAID 2014), and

*d* = margin of error.

A design effect of 1.5 was considered because multistage sampling was used (Amele et al. 2019). The calculated sample size assumes that the response rate would be 100%. However, there could be problems of nonresponse or withdrawals; to allow for this, the sample size was oversampled by 10%. Therefore, while the sample size for the study was 288, data were collected from 293 respondents. The number of respondents per site was calculated proportionally after dividing the sample size between the rural and urban areas.

### Data collection

A structured questionnaire was used as the research instrument for the survey. It was developed based on the study objectives and the literature review. The researcher adapted questions from different studies found in the literature. The structured questionnaire comprised the following subsections: demographics, knowledge about reproductive health services and available services, accessibility of YFHSs, experiences at the health facilities, barriers to utilisation of health services, factors that enhance the utilisation of YFHSs and strategies that could enhance the utilisation of YFHSs. This instrument comprised closed-ended questions. The questionnaire was translated into the local language (Chichewa), and prior to data collection, the researcher pretested the instruments with five youths at Gateway Clinic. Information collected at this clinic was not included in the final analysis of the research data and this health facility was not among the facilities for data collection for this study. After pretesting, ambiguous, sensitive, inappropriate and unnecessary words identified during pretesting were removed or modified. The required time for each respondent was also noted during pretesting, hence the questions were refined or modified to match the required time.

The recruitment process was for a period of 2 months from July to September 2023. The respondents were informed that participation was voluntary and an information sheet was read to each respondent before informed consent was signed. For youths younger than 18 years, an information sheet was read to parents or guardians of the youths, and parental consent was obtained through a signature or fingerprint on a parental consent form prior to the minor signing the assent form. The respondents were also informed that they were free to withdraw at any time, without any consequences. The information sheet about the study that had the researcher’s contact number in case of any follow-up questions was given to the respondents.

Using the structured questionnaire, data were collected from each person individually by the researcher as a supervisor with the assistance of four research assistants. The research assistants were trained to ask the questions precisely as stated in the instrument. Each data collector was required to conduct the survey on a maximum of 10 young people each day, with each survey taking about 30–40 min. The questions required the data collectors to circle available responses on the questionnaire. Data were collected from one site at a time until the collection was complete; the team then moved to the next site until the total required number of youth responses (*N* = 293) was reached. Data collection was completed in a total of 10 days. The respondents were informed that there was no payment for participating in the study, but transport was refunded for respondents who travelled more than 5 km.

### Data analysis

The principal researcher examined each questionnaire for completeness after which the data were entered into Microsoft Excel and then transferred to the Statistical Package for the Social Sciences (SPSS) version 26 for analysis. Descriptive data are presented through numbers, frequency distribution tables, percentages and proportions, thus providing a pictorial view of the study findings. Findings were also summarised using contingency tables to visually compare summarised data related to variables within the sample. To test the significance of the association between the variables under study, Chi-square (χ²) was used, and a statistical value of *p* < 0.05 was considered significant. Regression analysis was finally used to examine the influence of independent variables on the utilisation of YFHSs by youth in Blantyre.

### Ethical considerations

Prior to conducting this study, ethical approval was sought. The research proposal was reviewed by the UNISA Research and Ethics Committee which approved the research (NHREC Registration Number: Rec-240816-052; CREC Reference Number: 67129765_CREC_CHS_2021). The ethics approval is valid from 29 October 2021 to 29 October 2026. The research proposal was also submitted to the National Commission for Science and Technology of Malawi for ethical clearance and the study was approved (Ref No: NCST/RTT/2/6; Protocol No. P.08/22/663). Permission was also sought from the Blantyre District Health Office, the head office for all four health centres included in the study (Sibande & Malapela 2023).

Participation in the study was voluntary, and the respondents had the right to withdraw at any time without any consequence to themselves. Written informed consent forms, parental consent forms or assent forms were reviewed with each respondent and a signature, or fingerprint was obtained from all participants as well as from the parents of those below the age of 18 years. Anonymity of the participants was preserved by identifying each questionnaire with a number (Sibande & Malapela 2023). The signed consent and assent forms were kept separate from the questionnaires during the data collection process and were stored in separate compartments in a locked cupboard to ensure anonymity. All procedures during data collection were in accordance with the standards of UNISA and the National Commission for Science and Technology of Malawi research ethics committees and the 1964 Helsinki Declaration and its later amendments or comparable ethical standards (Sibande & Malapela 2023; Williams 2008).

### Rigour of the study

Content validity was ensured by incorporating ideas from similar studies in the literature review. Experts in reproductive health also analysed the instrument to assess its representativeness, appropriateness and adequacy of items representing the concept being tested. Suggestions from the experts were incorporated in the final instrument. Face validity of the instrument was enhanced by reviewing each question against the objectives of the study. The research instrument was also examined by experts in adolescent reproductive health and members of the research committees of UNISA and National Commission for Science and Technology of Malawi.

Four research assistants collected data in phase one; the researcher trained the research assistants on how to ask questions. Pretesting the structured questionnaire with five youths further ensured the reliability of the study. The pretesting of the instrument also assisted in the identification of anomalies and any necessary adjustments were made. Experts in adolescent reproductive health reviewed the structured questionnaire to ensure that it would be able to yield the same results on repeated measures. On the first day of data collection, the researcher and the four research assistants completed structured questionnaires on the same interview and checked the similarities and differences in the recordings. Corrections were made where necessary. The researcher also supervised the data collection process on an ongoing basis.

## Results

### Respondents’ demographic profile (*N* = 293)

A total of 293 respondents participated in the survey. Table 1 illustrates the sociodemographic characteristics of the respondents in the survey. More than half of the respondents (152 = 51.9%) were female. Most of the respondents were aged between 15 years and 19 years (143 = 48.8%), meaning that the majority were adolescents. Most respondents (136 = 46.6%) lived with both their parents, followed by those who lived with their mothers only (79 = 27%). Most were Christian (271 = 92.5%) and it was not surprising given the age of many respondents that most were students (170 = 58%). The next group was the unemployed (46 = 15.7%).

**TABLE 1:** Sociodemographic characteristics (*N* = 293).

Characteristics of respondents	Frequency	%
**Gender**		
Male	141	48.1
Female	152	51.9
**Age (years)**		
10–14	72	24.6
15–19	143	48.8
20–24	78	26.6
**Living with**		
Both parents	136	46.6
Mother only	79	27.0
Father only	3	1.0
Relatives	42	14.3
Friends	3	1.0
Others	30	10.2
**Religion**		
Christian	271	92.5
Muslim	22	7.5
**Occupation**		
Student	170	58
Business	39	13.3
Formal employment	36	12.3
Civil servant	2	0.7
Unemployed	46	15.7

The following conventions were adopted to present and discuss the quantitative research findings:

- *N* = Total sample
- *n* = Total of subvariables or topics

### Accessibility of Youth Friendly Health Services

**FIGURE 1:**
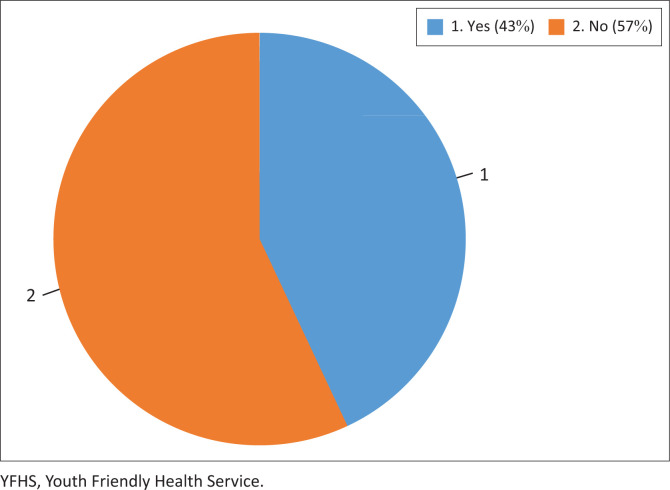
Distribution of respondents who have ever accessed Youth Friendly Health Services.

Figure 1 illustrates that less than half of the respondents had ever accessed YFHSs (43%).

### Knowledge about reproductive health services

Table 2 illustrates that two-thirds of the respondents in the current study already knew about YFHSs (194 = 66.2%), and among those who were aware of it, more than half of them had obtained this information either from friends (110 = 56.7%) or from nurses at health facilities (27 = 13.9%). The study found that just above half of the respondents (155 = 52.7%) also knew about the actual services offered at YFHSs. More than three-quarters of the respondents had previously received some education on SRH (229 = 78.2%). Knowledge on SRH issues would contribute to the utilisation of YFHSs where SRH services are also offered to the youth. There were two main sources of information for this, namely youth clubs (99 = 42.9%) and school (91 = 39.4%). Most of the participants preferred to receive information on SRH issues from nurses (134 = 45.7%), followed by their parents (60 = 20.5%). Regarding a signpost showing the availability of YFHSs at a health facility, more than half of the respondents (181 = 61.8%) had never seen any such signpost. The current study also found that two-thirds of the respondents had never received any printed educational materials such as leaflets on reproductive health from health facilities (192 = 65.5%).

**TABLE 2:** Respondent distribution by knowledge factors (*N* = 293).

Characteristics	Frequency	%
**Has heard of YFHSs**		
Yes	194	66.2
No	99	33.8
**Source of information of YFHSs (*n* = 194)**		
Friends	110	56.7
Parents or guardians	16	8.2
Nurse	27	13.9
Media	16	8.2
Community leaders	22	1.5
Others	3	-
**Any knowledge of services offered at YFHSs**		
Yes	155	52.9
No	114	47.1
**Has received any education on SRH**		
Yes	231	78.8
No	62	32.1
**Source of education on SRH**		
Community	10	4.3
Youth clubs	99	42.9
Health centre	25	10.8
Friends	3	1.3
Parents	3	1.3
School	91	39.4
**Preferred communicator about SRH issues**		
Parents	60	20.5
Family	16	5.5
Community	6	2.0
Grandparents	11	3.8
Friends	61	20.8
Nurse	134	45.7
**Has seen YFHS signpost**		
Yes	105	38.2
No	181	61.8
**Been given educational materials on** SRH		
Yes	11	34.1
No	192	65.5

YFHSs, Youth Friendly Health Services; SRH, sexual reproductive health.

### Experiences of the youth at Youth Friendly Health Services

Table 3 illustrates the experiences of the youth at YFHSs. Among the respondents who had previously accessed YFHSs, the majority (103 = 82.4%) agreed that they were given time to explain their problems to the health service providers. Most (102 = 81.0%) agreed that they were examined by the service provider and almost all the respondents who had ever accessed YFHSs (117 = 94.4%) agreed that they were treated respectfully.

**TABLE 3:** Distribution of respondents by experience at the Youth Friendly Health Services (*N* = 293).

Characteristics	Frequency	%
**Time given by the provider to explain (*n* = 125)**		
Yes	103	82.4
No	22	17.6
**Examined by provider (*n* = 126)**		
Yes	102	81.0
No	24	19.0
**Treated respectfully (*n* = 124)**		
Yes	117	94.4
No	7	5.6
**Rate providers’ character (*n* = 126)**		
Normal	38	30.2
Friendly	44	34.9
Professional	40	71.7
Harsh	1	0.79
Rude	3	2.3
**Treated with privacy (*n* = 126)**		
Yes	108	85.7
No	18	14.3
**Assured of confidentiality (*n* = 124)**		
Yes	101	81.5
No	23	18.5
**Provider explained or demonstrated (*n* = 125)**		
Yes	118	94.4
No	7	5.6

The service providers rated highly for positive characteristics, where 38 (30.2%) of the respondents said the providers were normal, 44 (34.9%) said the service providers were friendly and 40 (31.7%) said that they conducted themselves in a professional manner. Only 3% of the respondents rated the healthcare providers or YFHS focal personnel as harsh or rude. Most of the respondents said they were treated with respect to their privacy (108 = 85.7%) and 101 (81.5%) were assured of confidentiality. Almost all of the respondents accepted that the healthcare providers explained or demonstrated to them where necessary (118 = 94.4%). Thus, the current study indicates that respondents had no problems with the service providers.

### Barriers to utilisation of Youth Friendly Health Services

As is illustrated in Table 4, the study found that many of the possible barriers included in the current study’s survey were not barriers to utilisation of YFHSs. However, there were some possible barriers such as feeling shy to attend YFHSs (229 = 86.1%) and fear of being seen by relatives, parents or community members at the YFHS facility (230 = 87.1%). The majority of the respondents were satisfied with the YFHS working days (144 = 86.7%) and the operating times (148 = 93.1%). They were also satisfied with the YFHS outreach days (134 = 87.6%). Most of the respondents were happy with the cleanliness of the health centre environment (130 = 92.2%). It was also found that the greater part of the respondents did not need to be accompanied by anyone to attend YFHSs (220 = 88%) and did not mind seeing familiar faces at the facility (229 = 88.1%). The study also found that the cultural values and religious values for most of the respondents supported YFHS use (280 = 96.9%).

**TABLE 4:** Possible barriers to utilisation of Youth Friendly Health Services (*N* = 293).

Characteristics	Frequency	%
**Feels too shy to attend the YFHSs (*n* = 266)**		
Yes	37	13.9
No	229	86.1
**Fear of being seen at the YFHS facility (*n* = 264)**		
Yes	34	12.9
No	230	87.1
**Satisfied with YFHS working days (*n* = 166)**		
Yes	144	86.7
No	22	13.3
**Satisfied with YFHS operating time (*n* = 159)**		
Yes	148	93.1
No	11	6.9
**Satisfied with YFHS outreach days (*n* = 153)**		
Yes	134	87.6
No	19	12.4
**Health centre environment clean (*n* = 141)**		
Yes	130	92.2
No	11	7.8
**Needs company to access YFHSs (*n* = 250)**		
Yes	30	12
No	220	88
**Comfortable with seeing familiar faces at a YFHS facility (*n* = 260)**		
Yes	229	88.1
No	31	11.9
**Cultural value support YFHS use (*n* = 289)**		
Yes	280	96.9
No	9	3.1
**Religious value support for YFHS use (*n* = 289)**		
Yes	280	96.9
No	9	3.1

YFHSs, Youth Friendly Health Services.

### Statistical associations

#### Differentials in utilisation of Youth Friendly Health Services

This investigation included sociodemographic characteristics that included gender, age, who the young person was living with, religion and occupation. Other sections covered knowledge about reproductive health services and available services, accessibility of YFHSs, experiences at the health facilities, barriers to utilisation of health services, factors that enhanced utilisation of YFHSs and strategies that could enhance utilisation. Cross-tabulations and Pearson chi-square at 0.05 significance level and 95% confidence level were employed to determine the factors that had a significant influence on utilisation of YFHSs by youth in Blantyre.

As is illustrated in Table 5 at a 0.05 level of significance, the Chi-square test showed that the following variables had a significant association with utilisation of YFHSs (*p* < 0.05): gender, age, knowledge of services offered at YFHSs, ever received SRH education, ever seen a YFHS signpost or signage, ever been encouraged or ever been discouraged to access YFHSs, treated respectfully by the provider, issues demonstrated by provider, felt too shy to attend YFHSs and fear of being seen at YFHSs. The variables that were found to be significantly associated with accessibility of YFHSs (*p* < 0.05) were selected for further analysis.

**TABLE 5:** Differentials in the utilisation of Youth Friendly Health Services.

Characteristics	Yes	No	*n*	χ^2^	*p*
**Gender**				6.07	0.048
Male	52	89	141	-	-
Female	73	75	148	-	-
**Age (years)**				16.12	0.003
10–14	20	53	72	-	-
15–19	59	82	143	-	-
20–24	46	32	78	-	-
**Ever heard of services offered at YFHSs**				115.69	0.000
Yes	114	40	154	-	-
No	9	104	114	-	-
**Ever received SRH education**				16.57	0.000
Yes	111	228	229	-	-
No	14	45	59	-	-
**Ever seen YFHS signage or signpost**				49.22	0.000
Yes	71	31	102	-	-
No	51	130	181	-	-
**Given RH education materials**				32.99	0.000
Yes	64	35	99	-	-
No	60	131	191	-	-
**Ever been encouraged to access YFHSs**				420.59	0.000
Yes	117	42	159	-	-
No	8	119	127	-	-
**Ever been discouraged to access YFHSs**				26.45	0.000
Yes	55	31	76	-	-
No	66	129	195	-	-
**Treated respectfully by provider**				8.211	0.016
Yes	105	11	116	-	-
No	4	3	7	-	-
**Religious value support for YFHS use *n* = 289**				17.07	0.000
Yes	105	12	117	-	-
No	5	2	7	-	-
**Feels too shy to attend YFHSs**				6.80	0.033
Yes	10	27	37	-	-
No	113	114	228	-	-
**Fear of being seen at YFHSs**				6.75	0.034
Yes	9	25	34	-	-
No	114	115	229	-	-

Note: Significant association with utilisation of YFHSs (*p* < 0.05).

YFHSs, Youth Friendly Health Services; SRH, sexual reproductive health.

#### Predictors of Youth Friendly Health Service Utilisation

According to Table 6, females showed a strong statistical association with utilisation of YFHSs and had higher odds (adjusted odds ratio [AOR] 0.61, CI: 0.37, 0.98) of utilising YFHSs than male youth. Similarly, for age, those young people aged between 20 years and 24 years (AOR 0.19, CI: 0.06, 0.59) had higher odds of utilising YFHSs than those below 20 years.

**TABLE 6:** Predictors of Youth Friendly Health Service utilisation in the Blantyre disctrict.

Characteristics	Odds ratio				
	Unadjusted		Adjusted		*p*
	OR	95% CI	AOR	95% CI	
**Respondent’s gender**					
Male	1.00	-	1.00	-	1.000
Female	0.60	0.37, 0.96	0.61	0.37, 0.98	0.039*
**Respondent’s age (years)**					
10–14**RC**	1.00	-	1.00	-	1.000
15–19	0.53	0.29, 0.99	0.65	0.32, 1.35	0.245
20–24	0.27	0.13, 0.55	0.19	0.06, 0.59	0.001*
**Ever heard of services offered at YFHSs**					
No RC	1.00	-	1.00	-	1.000
Yes	33.54	12.05, 93.35	28.61	10.99, 74.45	0.000*
**Ever received an SRH education**					
No RC	1.00	1.00	1.00	-	1.000
Yes	3.09	1.59, 6.02	2.72	1.39, 5.32	0.002*
**Ever seen YFHS signpost**					
No RC	1.00	-	1.00	-	1.000
Yes	6.08	3.39, 10.92	5.58	3.14, 9.91	0.000*
**Given RH education materials**					
No RC	1.00	-	1.00	-	1.000
Yes	3.92	2.28, 6.74	3.74	2.17, 6.44	0.000*
**Ever been encouraged to access YFHSs**					
No RC	100	-	1.00	-	1.000
Yes	54.78	15.97, 187.92	52.26	15.41, 177.19	0.000*
**Ever been discouraged to access YFHSs**					
No RC	1.00	-	1.00	-	1.000
Yes	3.53	2.03, 6.14	3.41	1.94, 6.00	0.000*
**Treated respectfully by provider**					
No RC	1.00	-	1.00	-	1.000
Yes	7.22	1.34, 38.69	6.34	1.28, 31.43	0.009*
**Provider demonstrated or explained issues**					
No RC	1.00	-	1.00	-	1.000
Yes	3.53	0.60, 20.70	3.82	0.69, 20.94	0.045*
**Feels shy to attend YFHSs**					
No RC	1.00	-	1.00	-	1.000
Yes	0.37	0.17, 0.81	0.37	0.16, 0.83	0.012*
**Fear of being seen at YFHSs**					
No RC	1.00	-	1.00	-	1.000
Yes	0.36	0.15, 0.81	0.38	0.16, 0.88	0.020*

Note: The assessment was based on a logistic regression model where *n* = 293 and *p* = 0.05, **RC** denotes reference category, and

* denotes a significant category.

YFHSs, Youth Friendly Health Services; SRH, sexual reproductive health.

Likewise, youth who had heard about the services offered at YFHSs (AOR 28.61, CI:10.99, 74.45) and those who had previously received SRH education (AOR 2.72, CI:1.39, 5.32) had higher odds of utilising YFHSs than those who had never heard about YFHSs, the services offered and who had never received any SRH education, respectively.

Another predictor of utilisation of YFHSs was seeing a signpost or signage indicating the availability of YFHSs at a health facility. Those who had seen YFHS signage at a health facility had higher odds (AOR = 5.58, CI: 3.14, 9.91) of utilising it than those who had never seen any such signage.

Other predictors of utilisation of YFHSs were SRH education materials given at YFHSs and whether the young person was encouraged or discouraged from attending YFHSs. Those who received SRH education materials at the health facilities (AOR = 3.74, CI: 2.17, 6.44) and those who had ever been encouraged to attend YFHSs (AOR = 52.26, CI: 15.41, 177.19) or discouraged from attending (AOR = 3.41, CI: 1.94, 6.00) had higher odds of utilising YFHSs than their counterparts who never received any SRH education material at the health facility and those who had been neither encouraged nor discouraged to attend YFHSs, respectively.

Provider attitude was also found to be a predictor for the utilisation of YFHSs. Those who were treated with respect by the service provider (AOR = 6.34, CI: 1.28, 31.43), were assisted by a service provider and had providers who were able to demonstrate or explain issues (AOR = 3.82, CI: 0.69, 20.94) had higher odds of utilising YFHSs than those who were not treated respectfully or did not have issues demonstrated or explained, respectively.

How the youth themselves felt was also a predictor for utilising YFHSs. The study showed that youths who did not feel shy attending YFHSs (AOR = 0.37, CI: 0.16, 0.83) and those who did not fear being seen at YFHSs (AOR = 0.38, CI: 0.16, 0.88) had higher odds of utilising YFHSs than those who did feel too shy to attend or who feared being seen at YFHSs.

## Discussion

The aim of the study was to identify those factors that influence the utilisation of YFHSs in Blantyre, Malawi. This study revealed the underutilisation of YFHSs at a level of 43%.

Comparable results in a study by Violita and Hadi (2019) showed that only 24.3% of adolescents utilised YFHSs. Underutilisation of YFHSs was also identified in a study conducted in Ghana (Asare et al. 2020), where utilisation was at a level of 7.9% in Eastern Ethiopia indicating 25.3% (Sertsu et al. 2023) and in Northwest Ethiopia where utilisation of YFHSs was at a level of 28.9% (Tsegaw et al. 2023). A contrary finding was found in a study conducted in Nepal where more than two-thirds (67.5%) of the youths had utilised YFHSs at least once in the previous 12 months before the study (Sharma et al. 2023). The increased utilisation in Nepal was associated with high knowledge level about YFHSs by the youth, satisfaction from services provided, good behaviour of health workers, short waiting times, confidentiality by the service providers and good physical facilities among other reasons.

The results showed that female youths are more likely to utilise YFHSs than their male counterparts. This was supported by a previous study by Napit et al. (2020). Similarly, a previous study conducted in Southern Ethiopia found that utilisation of YFHSs was significantly associated with the female gender (Amaje et al. 2022). This can be attributed to the fact that it is the girl child who faces a greater number of effects from unintended pregnancies such as unsafe abortions, obstetric fistulae and infections, among other complications. Should more girls utilise YFHSs and access contraceptives, the number of unintended pregnancies and by extension unsafe abortions and associated complications would also reduce. The result also suggests the need for boys to be encouraged to attend YFHSs as leaving them behind would make the fight against unintended pregnancies futile.

The study has also shown that youths aged between 20 years – 24 years are more likely to utilise YFHSs in comparison to those below 20 years. This finding is consistent with the results of a study in Nepal where ages between 15 years and 19 years were positively associated with the utilisation of YFHSs (Napit et al. 2020). This result indicates the need for greater sensitisation among younger adolescents to encourage them to use YFHSs because it is the younger adolescent group that is more likely to face complications and any other negative results of unintended pregnancies.

This study found that knowledge about YFHSs and SRH issues are important factors in the utilisation of YFHSs. A study conducted in Uganda also found that a lack of SRH knowledge prevented the youth from accessing YFHSs (Musasizi 2023). Another previous study had similar findings where a lack of knowledge affected the behaviour of adolescents seeking SRH advice (Mattebo et al. 2019). This suggests that strategies need to be put in place to educate and sensitise more of the youth about the availability of YFHSs and SRH issues through school-based education as well as a greater sensitisation in the communities to increase awareness.

Seeing a signpost showing the availability of YFHSs at a health facility was a predictor for the utilisation of YFHSs. Unnoticeable signage was also found to be a factor that influenced the utilisation of YFHSs in a study from Ghana (Asare et al. 2020). This indicates the need for noticeable signage at the facilities informing the youth about YFHS availability at that facility, services that are offered and days and times when the services are offered written in languages that all the youth can understand.

Educational materials given at YFHSs were also found to be a predictor of the utilisation of YFHSs. This suggests the need for the Ministry of Health to produce printed educational materials on SRH and YFHSs to be given to the youth at the health facilities. These materials would reach other young people at home who may later opt to start using the YFHSs.

Provider attitude was also found to be another important factor of YFHS utilisation. Those who were treated respectfully and received demonstrations were more likely to continue using YFHSs. Likely, they would also recommend the services to their friends. This finding was like a previous study in Bangladesh (Ainul et al. 2020). A study in Nepal also showed that client satisfaction increased the utilisation of YFHSs (Sharma et al. 2023). However, many previous studies have revealed that service providers displayed negative attitudes towards the youth at YFHSs (Baigry et al. 2023; Mattebo et al. 2019). This suggests the need to train YFHS providers and only trained providers to be allocated to YFHSs.

Finally, the results showed that those youths who did not feel shy attending YFHSs and who did not fear being seen at YFHSs had higher odds of utilising YFHSs than those who did feel shy or feared being seen at YFHSs. This finding was also found in a previous study (Napit et al. 2020). This calls for the provision of dedicated spaces such as youth corners in health facilities.

The Health Belief Model was applied to the results. The Model asserts that an individual would take a preventive measure against a particular condition if it is associated with the individual’s perceived susceptibility to the condition, severity of the condition, benefits and barriers to taking the preventive action (Glanz et al. 2008:46). The model also asserts that there are cues that can trigger action, such as environmental events and media publicity. The study found that accessibility of YFHSs was still a problem as less than half of the respondents (43%) had ever accessed YFHSs. According to the Health Belief Model, this implies reduced perceived susceptibility and perceived benefits in utilising YFHSs among the youths. It also implies reduced perceived seriousness in not accessing YFHSs. Considering that more than two-thirds had heard about YFHSs, yet less than half had accessed the services, it implies reduced perceived seriousness in not utilising the services and low perceived benefits, according to the Health Belief Model.

Service providers were rated highly for positive characteristics. This suggests that the positive cues to action that service providers demonstrated could attract more youths to YFHSs according to the Health Belief Model. The study further found that cultural and religious values of the majority of respondents supported the use of YFHSs (96.9%). According to the Health Belief Model, the findings indicate reduced perceived barriers among the youth. This reflects the need to maintain the practices that support youths and address barriers so that more youths can attend YFHSs. Cues to action that encouraged the uptake of YFHSs included seeing a signpost for YFHSs and the availability of printed educational material for YFHSs such as leaflets. Lastly, self-efficacy to use YFHSs was facilitated by knowledge of YFHSs. According to LaMorte (2019:1), self-efficacy refers to the level of a person’s confidence in their ability to successfully perform a behaviour. In the present study, self-efficacy refers to the intention by the youth to utilise YFHSs.

### Implications of the study on the practice

#### Gender of the youth

Evidence from this study suggests that it is important to focus on both female and male youth when encouraging them to utilise YFHSs. Even though it is the girl who experiences a greater number of negative effects from unintended pregnancies, boys also ought to be knowledgeable about reproductive health issues to complement the prevention of unintended pregnancies. This calls for intensifying information giving about sexuality and SRH issues for boys at YFHSs, youth clubs, in schools and through mass media such as radio and television.

#### Focus on adolescents

The study suggests there is a need to increase the focus on adolescents so that more can utilise YFHSs. This group is more likely to face complications of childbearing should they succumb to unintended pregnancies. According to Abebe et al. (2020) globally, complications during pregnancy and childbirth are a second cause of death of 15–19-year-old girls. Considering that most adolescents are in school, there is a need for policymakers to make policies that increase information on SRH and YFHSs in primary and secondary schools.

#### Sexual reproductive health education and community sensitisation about Youth Friendly Health Services

The study suggests the need to intensify the provision of SRH education and sensitisation of the youth regarding the availability of YFHSs in different settings, including schools, youth clubs and the health facility in outpatient departments and in the communities. The use of mass media such as radio programmes and television programmes could also help in intensifying SRH education and sensitisation with assistance from the Ministry of Health in partnership with nongovernmental organisations that already focus on SRH.

#### Provider attitude

The results indicate that the provider’s attitude was positive. This suggests the need to encourage this through the deployment of trained service providers able to provide services to the youth with respect and in a nonjudgmental way in all the YFHS departments. All untrained service providers should be trained and refresher courses should be provided regularly.

### Implications for policy

#### School curriculum

The study indicates the need for primary and secondary school curricula to include information on the availability of YFHSs in the health facilities and encouragement for the youth to utilise these services. Results of this study will therefore be disseminated to the Ministry of Health responsible officers who could be fundamental in influencing change in policy that would increase utilisation of YFHSs.

#### Youth Friendly Health Service signpost

Evidence from this study suggests the need for clear and visible signpost showing the availability of YFHSs in health facilities. The signposts need to be in both English and any local languages so that the youth and others can easily read and know about the services that are available.

#### Printed sexual reproductive health materials

The study also suggests the need for the Ministry of Health to have printed materials such as leaflets on sexuality and YFHSs to be placed in the YFHS departments so that the youth can read while attending and some can be carried home to provide information on YFHSs and SRH issues to those who have never attended, thus encouraging them to attend YFHSs.

#### Dedicated space for Youth Friendly Health Services

The study suggests the need for dedicated space for the provision of YFHSs considering that privacy for the youth was being compromised when the services were offered on open space and together with adults. Some youths may feel shy or fear being seen at the YFHSs by people who might stigmatise them. This calls for health facilities to provide spaces such as youth corners or rooms dedicated to YFHSs only. This might help to increase the number of youths who attend YFHSs.

### Implications for future research

Future research is recommended to determine the friendliness of the YFHSs in Malawi.

## Limitations of the study

The generalisability of this study beyond Blantyre is limited owing to a small sample size and the study being conducted in one district only. However, the results of this study shed light on factors that influence the utilisation of YFHSs, and policymakers can benefit from it to improve the utilisation of the services.

## Conclusion

The findings of this study suggest that utilisation of YFHSs was still low (43%) and that different factors, including age, knowledge about YFHSs, signage for YFHSs, printed health education materials, provider attitudes, and barriers such as feeling shy or fear of being seen at the YFHSs, influenced utilisation of YFHSs. The study findings will help to fill in the gap in the provision of YFHSs and thus increase the utilisation of the services.
